# Cancer therapy-associated Takotsubo cardiomyopathy: A narrative review of mechanisms, drug associations, and clinical implications

**DOI:** 10.1016/j.ahjo.2026.100723

**Published:** 2026-01-15

**Authors:** Michael Simeon, Elizabeth Evans, Sally Arif, Thomas Granado, Tochukwu M. Okwuosa, Annabelle Santos Volgman, Salaheldin Abusin

**Affiliations:** a Rush Medical College, Chicago, IL, USA; b Division of Cardiology, Rush University Medical Center, Chicago, IL, USA; c Department of Pharmacy, Midwestern University College of Pharmacy and Department of Cardiology, Rush University Medical Center, Chicago, IL, USA; d Department of Internal Medicine, Rush University Medical Center, Chicago, IL, USA

**Keywords:** Takotsubo cardiomyopathy, Chemotherapy-induced cardiotoxicity, Cardio-oncology, Stress cardiomyopathy, Cancer-associated cardiomyopathy

## Abstract

Anticancer therapies have been increasingly associated with Takotsubo cardiomyopathy (TTC). As prior reports remain fragmented across case studies and drug-specific reviews, this paper offers one of the most comprehensive reviews to date of cancer therapy-associated TTC. While classically triggered by emotional or physical stress, TTC has been increasingly associated with chemotherapy and other cancer-directed therapies. This literature review explores the pathophysiology, clinical features, and evolving evidence linking anticancer agents to TTC, with a focus on cytotoxic cancer therapy, hormonal therapy, targeted treatments, and immune checkpoint inhibitors.

We describe the proposed mechanisms of cancer therapy-associated TTC, the diagnostic challenges, particularly in patients with cancer, and the complex management strategies, especially regarding the decision to resume oncologic treatment after TTC recovery.

Given the increasing use of cardiotoxic agents, a multidisciplinary approach to cardio-oncology care among patients with suspected TTC is crucial. More research is needed to understand the role of TTC in cardiac dysfunction among patients with cancer, clarify risk stratification methods, and improve outcomes for patients at risk of TTC during cancer treatment.

## 1 Background

Takotsubo cardiomyopathy (TTC), or stress-induced cardiomyopathy, is a transient left ventricular systolic dysfunction that mimics acute coronary syndrome but occurs without obstructive coronary disease [1]. It accounts for roughly 1–2% of suspected acute coronary syndrome cases and predominantly affects postmenopausal women [2]. While most data on TTC come from the general population, cancer patients appear to have a disproportionately higher risk due to emotional stress, critical illness, and exposure to cardiotoxic cancer therapeutic agents [3], [4]. Yet despite this growing recognition, evidence describing TTC in oncology patients remains limited and fragmented. This narrative review aims to synthesize the available evidence on the association between cancer therapeutic agents and TTC, summarize clinical features, discuss proposed mechanisms, and highlight management considerations in cancer therapy-induced TTC.

To compile the tables, a literature search was conducted using PubMed, Embase, Google Scholar, and Web of Science to identify English-language articles published between January 2010 and May 2025. Search terms included “Takotsubo Syndrome,” “Stress Cardiomyopathy,” “Apical Ballooning,” and individual drug names. Approximately 30 articles describing a confirmed or strongly suspected TTC diagnosis with a temporal association to a cancer therapeutic agent were included and summarized. Reports with insufficient clinical detail or alternative likely causes were not emphasized. Agent-level associations and proposed mechanisms are summarized in Table 1, while individual reported case characteristics, including clinical presentation, diagnostic findings, and outcomes, are summarized in Table 2.

**Table 1:** Cancer therapeutic agents associated with Takotsubo Syndrome: primary use, proposed mechanisms, and frequency of reported cases.

Type of drug category	Cancer drug	Primary usage	Mechanism of action in cancer	Associated mechanism in TTC	Reported cases	References
Cytotoxic (Alkylating Agent)	Carmustine	Treatment of brain tumors, lymphomas, and multiple myeloma	Alkylating agent that inhibits DNA/RNA synthesis, causing cell death through DNA cross-linking	Increased sympathetic tone, cytokine release, oxidative stress, myocardial damage	13	[20]
Cytotoxic (Antimetabolite)	5-Fluorouracil (5-FU)	Treatment of colorectal, breast, and head/neck cancers	Pyrimidine analog that inhibits DNA synthesis by disrupting thymidylate synthase	Coronary vasospasm, myocardial inflammation, ATP depletion	29	[24], [27], [28], [32], [39], [40], [41], [44], [45], [46], [52], [82], [83], [84], [85], [86], [87], [88], [89], [90], [91], [92], [93], [94], [95], [96], [97], [98], [99]
Cytotoxic (Anthracycline)	Daunorubicin	Treatment of leukemia, lymphoma	Intercalates into DNA, inhibiting topoisomerase II and causing DNA strand breaks	Direct myocardial injury, oxidative stress	3	[89], [100], [101]
Cytotoxic (Antimetabolite)	Cytarabine	Treatment of leukemia, lymphoma	Pyrimidine analog that inhibits DNA synthesis and promotes DNA chain termination	Direct myocardial injury, oxidative stress	3	[89], [102], [103]
Cytotoxic (Platinum Agent)	Oxaliplatin	Treatment of colorectal cancer	Platinum-based agent that forms DNA adducts, causing DNA crosslinking and inhibition of DNA replication	Neurotoxicity, autonomic hyperexcitation	7	[27], [32], [33], [41], [96], [97], [104]
Cytotoxic (Platinum Agent)	Carboplatin	Treatment of ovarian, lung, and breast cancer	Platinum-based agent that forms DNA crosslinks, inhibiting DNA replication	Neurotoxicity, endothelial dysfunction	5	[34], [35], [102], [105], [106]
Cytotoxic (Taxane)	Paclitaxel	Treatment of breast, ovarian, and lung cancer	Stabilizes microtubules, preventing their depolymerization, thus inhibiting cell division	Sympathetic activation, endothelial dysfunction	8	[34], [35], [52], [80], [102], [107], [108], [109]
Cytotoxic (Anthracycline)	Doxorubicin	Treatment of breast, lymphoma, leukemia	Intercalates into DNA, inhibiting topoisomerase II and generating reactive oxygen species that cause DNA damage	Myocardial toxicity, oxidative stress	4	[31], [100], [101], [108]
Cytotoxic (Vascular Disruptor)	Combretastatin	Treatment of solid tumors	Disrupts microtubule formation, inhibiting cell division and causing tumor cell death	Cellular dysfunction, DNA damage	1	[110]
Targeted Therapy (Anti-VEGF)	Bevacizumab	Treatment of colorectal, lung, and kidney cancer	Inhibits vascular endothelial growth factor (VEGF), preventing angiogenesis and tumor growth	Microvascular dysfunction, coronary artery spasm	2	[35], [111]
Targeted Therapy (EGFR Inhibitor)	Afatinib	Treatment of non-small cell lung cancer (NSCLC)	Inhibits epidermal growth factor receptor (EGFR) family receptors, interfering with signaling pathways that promote cell survival	Endothelial damage, myocardial stress	1	[51]
Targeted Therapy (EGFR Inhibitor)	Osimertinib	Treatment of epidermal growth factor receptor–mutant non–small cell lung cancer	Inhibits epidermal growth factor receptor mutations associated with non–small cell lung cancer, blocking tumor cell proliferation	Endothelial damage, myocardial stress	3	[35], [112], [113]
Targeted Therapy (HER2 Inhibitor)	Trastuzumab	Treatment of HER2-positive breast cancer	Monoclonal antibody targeting HER2, inhibiting signaling pathways that drive tumor cell proliferation in breast cancer	Cardiomyocyte dysfunction with reduced left ventricular ejection fraction	9	[52], [53], [54], [55], [75], [83], [114], [115]
Targeted Therapy (HER2 Inhibitor)	Pertuzumab	Treatment of HER2-positive breast cancer	Monoclonal antibody targeting HER2, inhibiting dimerization with other HER family receptors involved in tumor growth	Cardiomyocyte dysfunction, reduced left ventricular ejection fraction	3	[52], [115]
Immunotherapy (Checkpoint Inhibitor)	Pembrolizumab	Treatment of various cancers (e.g., melanoma, non–small cell lung cancer)	Programmed death-ligand-1 inhibitor that enhances immune responses by blocking interactions between PD-1 on T-cells and PD-L1 on tumor cells	Immune-mediated myocarditis, myocardial inflammation	7	[32], [54], [58], [102], [105], [112], [116]
Immunotherapy (Checkpoint Inhibitor	Ipilimumab	Treatment of melanoma, renal cell carcinoma, and others	Cytotoxic T-lymphocyte–associated protein 4 inhibitor that enhances T-cell activation, stimulating immune response against tumor cells	Immune-mediated myocarditis, myocardial inflammation	3	[50], [115], [116]

**Table 2:** Case reports of Takotsubo cardiomyopathy associated with chemotherapy.

Case Report/Article	Age, Sex, Comorbidities	Cardiac History (Y/N)	Suspected Culprit; Onset	Symptoms/Signs	Electrocardiogram/ Biomarkers	TTE Findings	CAG Findings	Clinical Course
Carmustine-Induced Takotsubo Cardiomyopathy	75-year-old male	Yes. Atrial fibrillation.	Carmustine: 12 h after 1st day of regimen.	Chest pain.	ST-segment depression in the lateral leads. Troponin level was 0.13 (reference <0.03). B-type natriuretic peptide level was not reported.	Ejection fraction was 30%, with global left ventricular hypokinesis and regional variation.	Nonobstructive coronary artery disease	Guideline-directed medical therapy initiated. Ejection fraction improved to 40% with improved wall-motion abnormalities at 2 days: subsequent deterioration after stem cell transplantation.
Oxaliplatin-Associated Takotsubo Cardiomyopathy in a Patient with Metastatic Gastric Cancer: Case Report	64-year-old woman with stage IV gastric adenocarcinoma and pulmonary and abdominal wall metastases.	No.	Oxaliplatin: during cycle 6, administered as monotherapy.	Oppressive chest pain with hypotension, bradycardia, and acute hypoxic respiratory failure.	ST-segment elevation in leads I and aVL. Troponin level was 75.71 (reference 0–40). B-type natriuretic peptide level was not reported.	Ejection fraction was 47%, with akinesis of the inferolateral wall and the mid and apical segments of the anterolateral wall.	No significant lesions	Intensive care unit admission. Cardiac magnetic resonance imaging at 7 days showed preserved systolic function. Twelve cycles of pembrolizumab completed without recurrent cardiovascular symptoms.
Takotsubo Cardiomyopathy After Oxaliplatin Chemotherapy Exposure: A Case Report	69-year-old woman with hypertension, hyperlipidemia, asthma, celiac disease, osteoporosis, long-term tobacco use, and cecal adenocarcinoma status post right colectomy.	Yes. Left ventricular ejection fraction of 60–65%.	Oxaliplatin: during infusion as monotherapy; initially suspected allergic reaction, with symptom worsening 12 h later.	Severe dyspnea.	Mild ST-segment changes evolved into diffuse T-wave inversion. High-sensitivity troponin increased from 192 to 206. B-type natriuretic peptide level was not reported.	Ejection fraction was 40–45%, with new hypokinesis of the mid to apical wall segments.	Normal	At 2 months, left ventricular ejection fraction was 45–50%, with mild improvement in apical hypokinesis.
Typical Takotsubo Cardiomyopathy in Patients with Cancer: Two Case Reports and a Literature Review	76-year-old woman with hormone receptor–positive breast cancer, hypertension, and hyperlipidemia.	Yes.	Tamoxifen: after 2 years of adjuvant therapy.	Admitted for suspicion of acute coronary syndrome.	ST-segment elevation in leads III, aVF, and V4–V6 with QT prolongation and flattened T waves; creatine kinase–MB isoenzyme 75 (upper limit of normal <24) and troponin I 9.85 (upper limit of normal <0.1); B-type natriuretic peptide not reported.	Ejection fraction 40% with apical ballooning and left ventricular outflow tract obstruction due to interventricular septal hypertrophy; Myocardial perfusion scintigraphy showed an irreversible defect.	Normal epicardial coronary segments with focal narrowing of the distal left anterior descending artery.	Tamoxifen discontinued with complete recovery of left ventricular function within 10 days; subsequently resumed and continued for 5 years without recurrence of Takotsubo cardiomyopathy.
Same as previous (Case 2)	75-year-old woman with hypertension, hyperlipidemia, impaired fasting glucose, obesity, and a history of endometrial cancer.	Yes.	External beam radiation: after 15 fractions over 3 weeks.	Admitted for suspected acute coronary syndrome.	T-wave inversion in leads V2–V6 with left axis deviation and a PR interval of 0.20 s. Creatine kinase–MB isoenzyme level was 19. Troponin T level was 0.178 (reference <0.014). B-type natriuretic peptide level was not reported.	Dyskinetic periapical ballooning of the left ventricle with preserved systolic function (ejection fraction 50%).	Normal, mildly tortuous epicardial coronary arteries.	Hospital admission followed by recovery of left ventricular function. Adjuvant radiotherapy continued without recurrence of Takotsubo cardiomyopathy
5-Fluorouracil–Induced Takotsubo Cardiomyopathy Complicated by Left Ventricular Thrombosis	42 yr female with remote history of smoking, recently given radiotherapy for anorectal cancer started on continuous 5-FU.	No.	5-FU: 7 days after radiotherapy, on day 5 of continuous 5-FU	Typical angina with dyspnea, cough, hallucinations, jugular venous distention, and tachycardia.	Sinus tachycardia with ST-segment elevation in the anterior leads and ST-segment depression in the inferolateral leads; troponin increased from 29 to 54 (reference <14); B-type natriuretic peptide 3650 pg/mL (reference <100).	Ejection fraction was 17%, with severe global hypokinesis, apical akinesis, and a matted apical thrombus.	Nonobstructive coronary artery disease	5-fluorouracil discontinued; uridine triacetate administered. Aspirin and apixaban given. Cardiac function normalized at 6 weeks (ejection fraction 70%) with thrombus resolution.
Afatinib-Related Takotsubo Cardiomyopathy in a Patient with Non–Small Cell Lung Cancer: Case Report	42-year-old woman with anorectal cancer and a remote history of tobacco use.	No.	Afatinib: after 19 four-week cycles; symptom onset 11 days after admission	Oppressive chest pain and dyspnea with jugular venous distention, S3 gallop, bilateral crackles, and tachycardia.	ST-segment elevation in anterior and septal walls with symmetric T-wave inversion (leads I, aVL, V4–V6) and corrected QT interval > 500 milliseconds; high-sensitivity troponin 0.147 (reference <0.03); B-type natriuretic peptide not reported.	Ejection fraction was 30%, with akinesis of all mid and apical segments and hypokinesis of the basal anteroseptal segment.	Not performed	
Takotsubo Cardiomyopathy During Anti-HER2 Therapy for Metastatic Breast Cancer	63-year-old woman with metastatic breast cancer.	No.	Pertuzumab and Trastuzumab.	Progressive dyspnea over 8–10 h with tachycardia.	Anterolateral ST-segment infarction. High-sensitivity troponin level was 719 (reference <15). B-type natriuretic peptide level was not reported.	Severely depressed left ventricular systolic function with apical dyskinesis and preserved basal contractile function.	Normal	Pursued palliative care and died about 1 month later
Chemotherapy-Induced Cardiomyopathy: A Case Report and Review of the Literature	45-year-old woman with locally advanced epidermoid carcinoma of the anal canal.	No.	5-Fluorouracil: onset the day after a 2-day induction course.	Nausea and vomiting with thoracic pain, followed by oppressive chest pain radiating to the right shoulder and cardiogenic shock.	ST-segment elevation of 1.5–2 mm in leads V4 and V5. Troponin T level was 348 (reference <14).	Ejection fraction was 30%, with apical and midventricular akinesis and preserved right ventricular function.	No coronary artery disease and no coronary vasospasm.	ICU admission with beta-blocker and angiotensin-converting enzyme inhibitor therapy. Left ventricular ejection fraction recovered to 60–65% within two weeks, and medications were discontinued. Chemoradiation completed with mitomycin C alone; 5-fluorouracil omitted
Fluorouracil-Induced Takotsubo Cardiomyopathy Causing Cardiogenic Shock: A Case Report of Clinical and Acute Cardiac Magnetic Resonance Imaging Findings	54-year-old woman with metastatic sigmoid adenocarcinoma, pituitary adenoma status post transsphenoidal surgery on long-term hormone replacement therapy, duodenitis, and hyperlipidemia.	No.	Fluorouracil: onset 1 day after combination chemotherapy with fluorouracil, leucovorin, and oxaliplatin.	Radiating chest pain with jugular venous distention and crackles; cardiogenic shock and acute hypoxic respiratory failure.	Tachycardia with T-wave inversion in leads V4–V6, I, and aVL. High-sensitivity troponin level was 679 ng/L. B-type natriuretic peptide level was not reported.	Severe global impairment of left ventricular systolic function (ejection fraction 10–15%) with mild functional regurgitation.	Not performed	Dual antiplatelet therapy; intensive care unit admission for high-flow oxygen and vasopressors. Cardiac magnetic resonance imaging at 4 days showed improvement; bisoprolol and angiotensin receptor blocker initiated. Imaging at 6 weeks showed resolution of acute findings.
5-Fluorouracil–Induced Acute Reversible Heart Failure Not Explained by Coronary Spasms, Myocarditis, or Takotsubo: Lessons from Magnetic Resonance Imaging	69-year-old patient with colon cancer.	No.	5-Fluorouracil: onset 3 days after treatment, following prior leucovorin and oxaliplatin.	Dyspnea and tachycardia with paroxysmal atrial fibrillation, low–normal blood pressure, and pulmonary edema.	Anterolateral ST-segment depression with T-wave inversion in leads I, aVL, and V2–V6; troponin 2540 (reference <49) and creatine kinase–MB isoenzyme 12 (reference <4); B-type natriuretic peptide not reported.	Left ventricular systolic function was globally depressed (ejection fraction 25%), with uniform hypokinesis of all segments and near-normal right ventricular function.	On day 3, there was no evidence of sclerosis, thrombosis, or vasospasm.	Treated with diuretics, nitroglycerin, beta-blocker, angiotensin-converting enzyme inhibitor, and mineralocorticoid receptor antagonist. Cardiac magnetic resonance imaging at 9 days showed normal left ventricular function (ejection fraction 60%) without dilation or wall-motion abnormalities.
Autopsy and Cardiac Magnetic Resonance Imaging Case of Bevacizumab-Related Cardiomyopathy	69-year-old with cervical cancer.	Yes. Ejection fraction 46% prior to chemotherapy.	Bevacizumab: onset 2 years after a 4-month course of bevacizumab and paclitaxel.	Congestive heart failure and new left bundle branch block.	New left bundle branch block with normal cardiac troponin levels and elevated N-terminal pro–B-type natriuretic peptide level (22,933 pg/mL).	Ejection fraction was 17%. Cardiac magnetic resonance imaging revealed linear mid-wall delayed enhancement in the basal to mid-septal wall.		Cardioprotective therapy without recovery of left ventricular ejection fraction. Death at one year; autopsy showed diffuse myocardial fibrosis.
Takotsubo Syndrome During Treatment With 5-Fluorouracil	Woman in her 40s with locally advanced rectal cancer.	No.	5-Fluorouracil: onset 2 days after starting continuous infusion.	Preceding nausea and vomiting, chest pain. Worsening radiating chest pain.	Mild ST-segment elevation in multiple contiguous leads (II, III, aVF, and V3–V6). Two hours later, T-wave inversion developed. Troponin I level was 239 (reference range 0–15).	Borderline left ventricular dilation with eccentric hypertrophy, hypokinesis extending from the mid inferoposterior and lateral walls to the apex, and moderately reduced systolic function.	Normal coronary ventriculography demonstrating apical hypokinesis with basal hyperkinesis.	Dual antiplatelet therapy, beta-blocker, and angiotensin-converting enzyme inhibitor initiated. Cardiac magnetic resonance imaging on day 5 showed left ventricular ejection fraction of 55%; beta-blocker discontinued. Transthoracic echocardiography at 3 weeks was normal. Tegafur/gimeracil/oteracil (S-1) with oxaliplatin replaced 5-fluorouracil; three cycles completed without complications.
Takotsubo Cardiomyopathy Caused by Infusion Reaction to Trastuzumab	65-year-old man with unresectable gastric cancer with lymphangitic carcinomatosis and respiratory failure.	No.	Trastuzumab: onset on day 7 during the first infusion, following prior oxaliplatin (day 1) and tegafur/gimeracil/oteracil for 6 days.	Fever, dyspnea, and chest pain.	T-wave inversion with ST-segment elevation in leads V3–V6; troponin I 0.219 ng/mL; creatine kinase 104 units/L and creatine kinase–MB isoenzyme 18; B-type natriuretic peptide not reported.	Apical akinesis with left ventricular hypokinesis and apical ballooning; ejection fraction 33.1%.	Not Performed	Chemotherapy discontinued with palliative diuresis. Left ventricular ejection fraction recovered to 56.1% at 12 days. The patient developed worsening hypoxia and respiratory distress and died at 23 days.
Takotsubo Syndrome as an Acute Cardiac Complication Following Combined Chemotherapy	61-year-old patient with hypertension, significant thromboembolic disease, and metastatic cervical cancer.	No.	Carboplatin and paclitaxel: onset 2 h after the third cycle.	Acute radiating chest pain with tachycardia.	ST-segment elevation in leads V2–V6 with T-wave inversion; troponin 0.824 (reference 0–0.014); B-type natriuretic peptide not reported.	Cardiac magnetic resonance imaging showed normal left ventricular size with moderate eccentric hypertrophy, apical and midventricular hypokinesis, ejection fraction 38.3%, and no early or late enhancement.	No obstructive lesion	Chemotherapy discontinued; beta-blocker, angiotensin-converting enzyme inhibitor, sodium–glucose cotransporter 2 inhibitor, and anticoagulation initiated. Left ventricular function normalized at 4 weeks.

Abbreviations: HER2, human epidermal growth factor receptor 2; ICU, intensive care unit.

a. Definition, triggers, and epidemiology

The term “takotsubo” originates in Japan and refers to the distinctive ballooning shape of the left ventricle, which resembles an octopus trap [5]. This form of cardiomyopathy features a narrow neck and rounded apex, seen in the majority of cases. Data from the International Takotsubo Registry, which analyzed 1750 patients across the United States and Europe, further emphasizes that the condition, as previously mentioned, predominantly affects women (89.8%) with a mean age of 66.8 years. While emotional triggers accounted for 27.7% of cases, 29% had no identifiable trigger at all, and physical stressors were identified in 36% of patients [1]. Although rare, TTC has also been reported following COVID-19 vaccination, suggesting a temporal link potentially mediated by sympathetic activation and catecholamine surge [6]. Acute emotional stressors have been shown to induce brain activation, increasing the bioavailability of cortisol, epinephrine, and norepinephrine [7], [8].

b. Clinical features

Clinically, TTC patients typically present with sudden chest pain, dyspnea, or syncope, closely resembling acute coronary syndrome [1]. TTC accounts for approximately 2% of patients hospitalized with suspected acute coronary syndrome, underscoring its clinical relevance and diagnostic challenge. The higher incidence in postmenopausal women (5.2 per 100,000) compared to men (0.6 per 100,000) highlights potential hormonal influences in its pathogenesis. Although the prognosis is generally favorable with a low mortality rate (1–3%), a subset of patients may experience recurrent episodes or high-risk features associated with poorer outcomes [8], [9].

A comprehensive review reported that the most common symptoms of TTC are acute chest pain (in >75% of cases), followed by shortness of breath (∼50%), dizziness (>25%), and syncope (5–10%) [4]. On physical examination, patients may also present with signs of hemodynamic compromise such as respiratory distress, hypotension, narrow pulse pressure, and an S3 gallop. Additional findings may include jugular venous distention, pulmonary crackles, a systolic ejection murmur due to left ventricular outflow tract obstruction (LVOTO) or mitral regurgitation, and, in some cases, lower extremity edema [10], [11].

The most common anatomical variant of TTC is apical ballooning, seen in 75–80% of cases, characterized by apical akinesis and basal hyperkinesis. Medina de Chazal et al. describe less common variants, including midventricular (10–20%), basal/inverted (<5%), and rare forms such as biventricular, right ventricular, and focal types. These variants differ in severity and hemodynamic impact, though the reasons for this variability remain unclear [8], [10]. A multinational analysis from the International Takotsubo Registry (*n* = 3957) spanning 2004 to 2021 revealed shifting trends in TTC presentations across Europe and the U.S. First, the proportion of male patients increased from 10% to 15% over the study period, indicating a growing recognition of TTC in men. Second, physical triggers became more prevalent, rising from 39% to 58% (*p* < 0.001). Emotional triggers became relatively less common [12]. Lastly, the incidence of midventricular TTC increased from 18% to 28% (*p* = 0.018), suggesting an improvement in the detection of non-apical variants. However, apical ballooning remains the most common variant [13].

Several diagnostic criteria exist for TTC, including those of the Heart Failure Association of the European Society of Cardiology [2], InterTAK [14], Diagnostic Criteria and Revised Mayo Clinic Criteria [15],—all of which emphasize transient left ventricular dysfunction, absence of obstructive coronary disease, new electrocardiogram (ECG) or biomarker changes, and exclusion of alternate diagnoses such as myocarditis or pheochromocytoma [2], [14], [15].

c. Pathophysiology

While the exact pathophysiology remains unclear, a leading hypothesis suggests that stress-induced catecholamine surges result in myocardial stunning and microvascular dysfunction [16], [17]. Other proposed mechanisms include coronary microvascular dysfunction, which may reduce myocardial perfusion without obstructive coronary artery disease, and estrogen deficiency, which could explain the higher prevalence of TTC in postmenopausal women [18].

Medina de Chazal et al. highlight the central autonomic nervous system (CANS) as a key regulator of stress-induced myocardial dysfunction in their systematic review [10]. Their findings, supported by neuroimaging studies, demonstrate increased cerebral blood flow in the hippocampus, brainstem, and basal ganglia during acute TTC episodes, suggesting the role of stress-induced neural activation. This phenomenon is similar to “neurogenic stunned myocardium,” previously observed in patients with acute stroke, seizures, or electroconvulsive therapy. In response to extreme emotional or physical stress, activation of brainstem and hypothalamic centers triggers a surge in sympathetic nervous system output, leading to the spillover of norepinephrine and neuropeptide Y (NPY) from sympathetic nerve terminals into the myocardium. This excessive release can induce direct cardiotoxicity and promote epicardial and microvascular dysfunction, ultimately contributing to the transient ventricular impairment seen in TTC [10].

## 2 Relationship between cancer therapy and TTC

Cancer therapy-induced cardiotoxicity (CIC), including TTC, has been increasingly recognized as a significant concern in cancer patients. The cardiotoxic effects of cancer therapy can manifest acutely or in a delayed fashion, with some patients developing heart failure or arrhythmias months or years after treatment. The relationship between cancer therapy and cardiovascular complications is complex, involving both direct myocardial toxicity and indirect mechanisms such as vascular injury or inflammation, [20] and has garnered increasing attention, particularly concerning cancer therapy-induced TTC. There is emerging evidence linking a range of cancer therapeutic agents implicated in TTC, such as cardiotoxic cancer therapy agents, immune checkpoint inhibitors (ICIs), hormonal therapy, targeted therapy, and radiation therapy. Specific cancer therapeutic agents, such as the anti-metabolite 5-fluorouracil, have been implicated in triggering TTC, possibly due to coronary vasospasm or direct myocardial toxicity [2], [21], [22]. Notably, ICIs, including agents targeting the CTLA-4 and PD-1/PD-L1 pathways, have revolutionized cancer treatment but are associated with immune-related adverse events that affect various organ systems, including the cardiovascular system [23], [24], [25]. Given the increasing use of ICIs and other agents in oncology, understanding cancer therapy-induced TTC is crucial for early recognition and management [20], [26].

## 3 Pathophysiology & proposed mechanisms of cancer therapy-associated TTC

### 3.1 Cancer therapy and TTC

Cancer therapy-associated TTC has been described across multiple drug classes, though nearly all available evidence comes from isolated case reports rather than controlled studies. Cytotoxic agents—including 5-fluorouracil (5-FU), anthracyclines, antimetabolites, platinum compounds, and combination regimens such as carboplatin and paclitaxel have been temporally linked to TTC in the literature [23], [24], [25], [26], [27], [28], [29], [30], [31], [32], [33], [34], [35], [36], [37], [38], [39]. Proposed mechanisms reflect established TTC physiology and include coronary vasospasm, microvascular dysfunction, autonomic dysregulation, oxidative stress, and myocardial stunning. Among these agents, 5-FU has the most frequently reported association, with TTC episodes hypothesized to arise from vasospasm, microvascular injury, and energy depletion within cardiomyocytes [21], [38], [39], [40], [41], [42], [43], [44], [45], [46], [47].

Anthracyclines such as doxorubicin may predispose patients to TTC by generating reactive oxygen species, causing mitochondrial injury and myofibrillar disruption, while platinum agents like oxaliplatin may influence TTC risk through autonomic imbalance, peripheral neurotoxicity that affects cardiac regulation, or ion-channel alterations [28], [29], [30], [31], [32]. Because TTC can also be triggered by cancer-related physiological stressors—including systemic inflammation, anemia, pain, metabolic derangements, or psychological distress—causality cannot be assigned to any individual drug.

### 3.2 Other targeted cancer therapeutics and TTC

Targeted therapies, which are cancer drugs that block specific molecular pathways involved in tumor growth, and immunotherapies have also been linked to TTC through small, published reports. VEGF pathway inhibitors (bevacizumab, axitinib, sunitinib) and EGFR inhibitors (afatinib, osimertinib) may promote TTC via microvascular injury, endothelial dysfunction, or autonomic imbalance, mechanisms that align closely with TTC's established pathophysiologic triggers [22], [48], [49], [50], [51], [52], [53], [54], [55], [56].

Endocrine therapies such as tamoxifen, a selective estrogen receptor modulator, have also been linked to rare TTC cases, with reports suggesting that estrogen suppression may heighten catecholamine sensitivity and microvascular vulnerability in predisposed post-menopausal women [57]. TTC has also been described with HER2-directed therapies such as trastuzumab and pertuzumab, with proposed pathways involving impaired myocardial resilience to catecholamine surges when HER2-related signaling is disrupted [51], [52], [53], [54].

Immune checkpoint inhibitors (ipilimumab, nivolumab) may rarely precipitate TTC through T-cell–driven cytokine-mediated inflammation, creating a state of heightened physiologic stress that is conducive to TTC [56], [58], [59]. Given the absence of prospective studies and the reliance on anecdotal case reports, these associations should be interpreted cautiously. Table 1 summarizes cancer therapeutic agents reported to be associated with TTC, along with hypothesized TTC-specific mechanisms described in the literature.

## 4 Multifactorial etiology and diagnostic challenges in TTC in patients with cancer

While certain anti-neoplastic drugs have known associated cardiotoxicity, no study to date has clearly linked TTC to any particular drug [60]. A history of cancer itself carries a higher risk of TTC [9], [61]. The increased incidence of TTC in cancer patients and multifactorial etiology suggest that multiple factors make the cancer patient uniquely vulnerable to this phenomenon. Before attributing TTC to the anti-neoplastic regimen, the clinician must consider other potential triggers.

### 4.1 Diagnosis

Precise diagnosis of TTC can be challenging. The clinical presentation overlaps substantially with acute coronary syndrome as well as other cardiomyopathies, and up to one-third of cases lack an identifiable trigger [8]. A patient's cancer history broadens the differential diagnosis, given the various cardiotoxicities associated with anti-neoplastic agents. Cancer-related physiological stress, such as systemic inflammation, elevated catecholamines, metabolic derangements, anemia, pain, and psychological distress, has been recognized as a trigger in large TTC registries, including the International Takotsubo Registry. These factors, combined with comorbid conditions common in oncology patients (infection, renal dysfunction, electrolyte abnormalities), may lower the threshold for TTC even in the absence of cardiotoxic therapies [1], [2], [5]. Cancer history should simultaneously raise the index of suspicion for TTC, given predisposing factors unique to cancer patients. Beyond diagnostic challenges, the appropriate management of anti-neoplastic therapy in patients who develop TTC is uncertain, with no specific guidelines to date.

Whenever TTC is suspected, it is imperative to rule out acute coronary syndrome given the time-sensitive nature of revascularization. Both syndromes commonly present with chest pain and/or dyspnea. An initial ECG may show ST elevations. In contrast to ST-elevation myocardial infarction (STEMI), the ECG in TTC often lacks reciprocal ST depressions, although it may exhibit diffuse T-wave inversions. The echocardiogram in TTC classically shows basal to mid-ventricular hyperkinesis with apical ballooning [8]. While focal phenotypes have been described, wall motion abnormalities in TTC generally span beyond a single coronary perfusion territory [8]. Other clues that point toward TTC rather than acute coronary syndrome include right ventricular involvement, higher B-type natriuretic peptide elevation, and mild troponin elevation, which is disproportionate to the degree of left ventricular dysfunction [62]. However, these markers can also be abnormal/elevated among patients experiencing cancer therapy-associated cardiac dysfunction [60]. While coronary disease and TTC are not mutually exclusive, elucidation of coronary disease on angiogram that does not explain the acute onset of cardiomyopathy strengthens the diagnosis of TTC [8].

### 4.2 Triggers

Medical stress in various forms can precede TTC. Sepsis is a well-documented trigger. Lozahic et al. propose a two-hit model in cancer patients, in which the immunocompromised state, combined with a systemic inflammatory response (e.g., from COVID-19), leads to cardiac complications. They attributed these complications to the virus's ACE2 downregulation, IL-6, other cytokines, endothelial dysfunction, and thromboembolic phenomena [63].

Anemia-induced myocardial hypoxia may be another factor that increases vulnerability in the cancer patient. Numerous cytotoxic agents cause anemia due to bone marrow suppression and worsening renal function [64]. Baik et al. propose that anemia in the setting of high-cardiac-output states, such as sepsis, tumor lysis syndrome, or cytokine release syndrome, can exacerbate hypoxia in cancer patients [64].

Systemic cancer therapy or radiotherapy to the thoracic region may cause coronary endothelial dysfunction [7]. Other medical stressors to consider in the cancer patient are anesthesia, surgery, cancer pain, or even the stress of the cancer diagnosis. In the general population, patients with TTC have more cardiovascular risk factors at baseline [1]. Thus, it is plausible that cardiac co-morbidities mediate the risk of TTC or other cardiotoxicities in the cancer patient.

## 5 Management & prognosis

### 5.1 Overall consideration for the management of TTC in the cancer patient

The management of cancer therapy-associated TTC is guided almost entirely by case reports and small observational studies [1], [62]. Treatment principles, therefore, mirror those used in the general TTC population, with modifications based on oncologic status, treatment-related toxicities, and comorbidities.

The cancer patient is overall quite vulnerable to developing TTC given various medical and psychosocial stressors that create a high catecholamine state, treatment-mediated mechanisms, and baseline cardiac co-morbidities. When TTC follows anti-neoplastic treatment, the patient and clinician are left with a management dilemma due to the inability to pinpoint the trigger with certainty. In a small retrospective cohort study of cancer patients at MD Anderson Cancer Center who developed TTC, a majority of patients were able to safely resume anti-neoplastic therapy after recovery of the left ventricular ejection fraction without relapse of TTC. In contrast, the literature on 5-FU generally does not encourage reinstatement of 5-FU therapy after restoration of cardiac function, given high rates of further cardiac complications in patients already affected by cardiotoxicity [60]. Since there are no large-scale studies or guidelines to support decision-making in this scenario, the decision whether to adjust anti-neoplastic therapy after TTC may be made on a case-by-case basis, conveying the honest uncertainty of the exact etiology to the patient and using shared decision-making.

### 5.2 Acute settings

Acute management typically includes supportive care and guideline-directed heart failure therapy when hemodynamically tolerated. Beta-blockers and ACE inhibitors are often used to reduce sympathetic drive and afterload, though evidence for benefit in cancer therapy-induced TTC is limited to case reports and registry data [65], [66], [67], [68]. Careful dosing is required because cancer patients frequently have hypotension, renal dysfunction, and treatment-related cytopenias [1].

In severe presentations, including cardiogenic shock, management must be individualized. Up to 10% of TTC cases may develop shock, and LVOT obstruction must be recognized early because inotropes can worsen obstruction [1], [69]. Case reports support a cautious approach to beta-blockers in LVOTO and the use of temporary mechanical circulatory support when needed [70], [71].

### 5.3 Thromboembolic risk

Thromboembolic risk is higher in TTC patients with malignancy due to both apical ballooning and cancer-associated hypercoagulability. This includes the documented incidence of left ventricular thrombus in TTC (1.3% in the International Takotsubo Registry and up to 2.5% in GEIST) [1], [70], [72]. Anticoagulation is considered when a left ventricular thrombus is present or strongly suspected [73], but bleeding risk is significant in patients receiving myelosuppressive cancer therapy; therefore, therapy must be individualized [34], [74], [75].

### 5.4 Long-term considerations

Long-term considerations emphasize close follow-up during the first 4–12 weeks, when LV function typically normalizes [76], [77]. Cancer treatment may be cautiously resumed once cardiac function stabilizes, and recurrence of TTC due to cancer therapy appears uncommon [25]. An interdisciplinary cardio-oncology evaluation is recommended for risk stratification and decisions around treatment re-initiation [5], [78], [79], [80].

Overall, management of TTC in cancer patients remains poorly defined, with limited data guiding treatment decisions [81]. Most recommendations are extrapolated from non-cancer TTC populations, highlighting the need for prospective studies in this high-risk group.

## 6 Future directions & research gaps/limitations

Despite growing awareness of cancer therapy-induced TTC, important gaps remain. Most current data are limited to case reports and retrospective series, showing the need for prospective studies to clarify incidence, risk factors, and outcomes specifically in the cancer population [1]. The role of biomarkers, such as B-type natriuretic peptide, troponins, and inflammatory markers, in the early detection or prediction of TTC warrants further exploration. Similarly, preventive strategies, including the potential use of beta-blockers, statins, or antioxidants, have not been rigorously tested in this population. Genetic susceptibility also remains poorly understood; identifying patient-specific risk factors could help refine screening and prevention efforts. Finally, the absence of standardized diagnostic and management guidelines contributes to variability in clinical practice, underscoring the need for consensus-driven, evidence-based protocols.

## 7 Conclusion

TTC represents a unique intersection of cardiology and oncology, where physiological vulnerability, pharmacologic insult, and psychological stress converge. While traditionally associated with emotional or physical stress, emerging evidence now implicates a wide range of cancer therapies, including cytotoxic agents, endocrine therapies, targeted treatments, and immune checkpoint inhibitors, as potential triggers. These agents may induce TTC through distinct yet overlapping mechanisms such as sympathetic overactivation, coronary vasospasm, endothelial dysfunction, and direct myocardial toxicity. Cancer patients are particularly at risk due to their baseline cardiovascular burden, cumulative treatment-related insults, and the systemic stressors imposed by their disease.

The diagnosis of cancer therapy-associated TTC remains challenging, as TTC often mimics acute coronary syndromes and lacks pathognomonic features. Standardized diagnostic criteria help guide evaluation; however, clinical judgment remains essential, especially in oncology settings where confounding cardiac pathologies are common. Management strategies should be individualized, focusing on hemodynamic support, prevention of thromboembolic complications, and careful consideration of whether to continue or modify cancer therapy.

Despite the growing recognition of cancer therapy-associated TTC, high-quality data to guide prevention, long-term monitoring, and treatment decision-making are limited. A multidisciplinary approach, involving both cardiology and oncology teams, is essential to balance oncologic efficacy with cardiovascular safety. Continued research is needed to better elucidate and understand this phenomenon among patients with cancer, and to develop evidence-based guidelines for risk stratification, recurrence prevention, and the safe reinitiation of therapy in affected patients.

## CRediT authorship contribution statement

**Michael Simeon:** Conceptualization, Writing – original draft, Writing – review & editing. **Elizabeth Evans:** Writing – original draft. **Sally Arif:** Writing – original draft. **Thomas Granado:** Writing – original draft. **Tochukwu M. Okwuosa:** Writing – original draft, Writing – review & editing. **Annabelle Santos Volgman:** Writing – review & editing. **Salaheldin Abusin:** Writing – review & editing.

## Ethical statement

This manuscript is a narrative review of previously published literature and did not involve human participants or animal subjects. Ethical approval was therefore not required.

## Declaration of Generative AI and AI-assisted technologies in the writing process

ChatGPT-5 (OpenAI, San Francisco, CA, USA) was used to assist with language editing and table formatting. All AI-generated suggestions were reviewed and verified by the authors, who take full responsibility for the content.

## Declaration of competing interest

None.
